# A novel *G6PD* gene variant in a Chinese girl with favism

**DOI:** 10.1002/jcla.23402

**Published:** 2020-06-17

**Authors:** Shanshan Shen, Qian Xiong, Wenqian Cai, Hao Xiong, Xijiang Hu

**Affiliations:** ^1^ Wuhan Children’s Hospital (Wuhan Maternal and Child Healthcare Hospital) Tongji Medical College Huazhong University of Science & Technology Wuhan City China

**Keywords:** acute hemolytic anemia, China, favism, G6PD deficiency, *G6PD* variant

## Abstract

**Background:**

Glucose‐6‐phosphate dehydrogenase (G6PD) deficiency is the most common human enzymopathy. The human *G6PD* gene is highly polymorphic, and over 200 mutations have been identified, many of which are associated with hemolytic anemia. Here, we analyzed the clinical genetics data of a Chinese girl with favism who developed acute hemolytic anemia after fava bean ingestion.

**Methods:**

The clinical genetics data of the proband who developed acute hemolytic anemia were collected and analyzed, and *G6PD* gene exons were sequenced in the proband and her family.

**Results:**

We reported for the first time a novel *G6PD* gene variant in a Chinese girl, which we named “G6PD Wuhan.” This variant is localized exon 3 of the *G6PD* gene at genomic position 141G > C, that is a change from p.Lys47 to Asn. The bioinformatics analysis and protein modeling study indicated this variant may have negative effects on the enzyme activity of G6PD.

**Conclusions:**

Our results indicated that favism in the proband was caused by this novel heterozygous variant (c.141G > C) in *G6PD*. The variant in *G6PD* has implications for genetic counseling and could provide insights into the functional roles of *G6PD* mutations.

## Graphical abstract

A novel variant (c.141G > C) in the exons of the *G6PD* gene may have negative effects on the enzyme activity of G6PD.

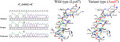

## 1 INTRODUCTION

Glucose‐6‐phosphate dehydrogenase (G6PD) is a metabolic enzyme that catalyzes the oxidation of glucose‐6‐phosphate to 6‐phosphoglucono‐δ‐lactone concomitant with the reduction of nicotinamide adenine dinucleotide phosphate (NADP) to NADPH, which is the first step in the pentose phosphate pathway. It plays a key role in protecting cells against oxidative stress by regulating the level of reduced glutathione.^1^ G6PD deficiency is a hereditary genetic defect and is one of the most prevalent enzymopathies in humans, which affects more than 400 million people worldwide.^2^ Several mutations in the *G6PD* gene could affect the stability and activity of the enzyme, which induced irreversible oxidative damage in the red blood cells (RBCs) and result in a wide spectrum of clinical manifestations.^3^, ^4^ G6PD deficiency can be asymptomatic or display severe clinical manifestations such as favism, hemolytic anemia, chronic nonspherocytic hemolytic anemia (CNSHA), and neonatal hyperbilirubinemia, which is closely linked to neonatal kernicterus that can lead to death.^5^

The *G6PD* gene is located in the X chromosome (Xq28) and includes 13 exons and 12 introns, spanning approximately 18.5 kb.^6^ Therefore, genotypes in males are hemizygous (G6PD normal/deficient), while in females are heterozygous or homozygous (G6PD normal/deficient). In heterozygous females, the G6PD activity is subject to the phenomenon of X‐chromosome inactivation. Currently, nearly 400 *G6PD* variants have been discovered based on biochemical diagnosis, among which 217 *G6PD* mutations have been identified at the DNA level.^7^, ^8^ Most of the mutations reported are point mutations, which cause single amino acid substitutions. In China, approximately 35 different molecular abnormalities of G6PD genotype have been identified.^9^, ^10^, ^11^

In the present case, the clinical genetics data of a family with G6PD deficiency were collected and analyzed. In this family, we identified a novel variant in *G6PD* gene in a 2.4‐year‐old girl with favism. Structural analysis and the potential effects of this variant on the enzyme structure or activity were also analyzed using in silico tools.

## 2 MATERIALS AND METHODS

### 2.1 Patients

A 2.4‐year‐old girl with acute hemolytic anemia was referred to Wuhan Children's Hospital, and the patient was diagnosed with favism when she suffered a hemolysis crisis with severe anemia after ingesting fava beans. The biochemical and blood markers were assayed. The patient and her parents were included in the study.

### 2.2 Ethics statements

This study was approved by the Ethics Committee of the human subjects at “Wuhan Children's Hospital.” Informed consent for molecular and biochemical studies was obtained from the patient's parents before collecting blood samples. The proband and her parents are all Han Chinese from Hubei Province, China.

### 2.3 Enzyme analyses

Enzyme activity measurement was performed 6 months after acute hemolytic anemia episode to ensure the assessment would be at a steady‐state representation of G6PD activity. The G6PD activity was performed on the proband and her parents. The analysis for G6PD deficiency was performed using the specific G6PD mensuration reagent kit (Guangzhou Fenghua Co. Ltd., China), and the enzyme activity was expressed as the ratio of G6PD/6PGD. The normal G6PD enzyme activity was recognized as G6PD/6PGD* > *1.0; G6PD/6PGD < 1.00 was identified as a deficient activity.

### 2.4 Genomic analysis

Genomic DNA was collected from peripheral blood leukocytes using the QIAamp DNA Blood Mini Kit (Qiagen, Germany) according to the manufacturer's instructions. DNA sequencing of the 12 coding exons and intronic flanking regions of the *G6PD* gene was performed using the Sanger method with the ABI PRISM^®^ 3130 automated capillary sequencer as previously described.^12^ RefSeq.NM_001042351.2 was used as the reference sequence. GeneScan Analysis Software was used to analyze the sequencing results.

### 2.5 In silico analysis

The potential effect of this mutation on the enzyme structure was analyzed using Sorting Intolerant From Tolerant (SIFT) and MutationTaster. For structural analysis, the X‐ray structure of G6PD deposited in the Protein Data Bank was applied (PDB code 2BHL and 2BH9), and a p.Asn47 mutated protein model was created with SWISS‐MODEL and visualized using Swiss PDB Viewer.

## 3 RESULTS AND DISCUSSION

The data of G6PD activity and hematological profile of the patient are described in Table 1. The proband developed severe acute hemolytic anemia after eating fava beans (hemoglobin level reached the critical value of 47 g/L) and treated with blood transfusion. The proband's mother had normal G6PD activity (G6PD/6PGD as 1.96); however, the proband's father presented with moderately deficient activity (G6PD/6PGD = 0.38, 38% residual activity) and displayed asymptomatic status. The father reported that he had no significant problems after eating fava beans.

**Table 1:** Hematological profiles and G6PD activity of the proband

Markers	Proband	Normal range
Sex/age (years)	F/2.4	
RBC (×10^12^/L)	1.65	3.7～5.3
HGB (g/L)	47	110～149
HCT (%)	15.2	35～47
MCV (fL)	92.1	80～98
MCH (pg)	28.5	26～31
MCHC (g/dL)	309	300～350
TBIL(umol/L)	74.8	3.0～22.0
BU(umol/L)	61.3	0～19.0
ALT(U/L)	7	9～52
AST(U/L)	57	15～46
G6PD/6PGD	0.65	>1

Abbreviations: ALT, alanine aminotransferase; AST, glutamic oxaloacetylase; BU, unconjugated bilirubin; HGB, hemoglobin; MCH, mean hemoglobin content; MCHC, mean corpuscular hemoglobin concentration; MCV, mean cell volume; RBC, red blood cells; TBIL, total bilirubin.

**Figure 1:**
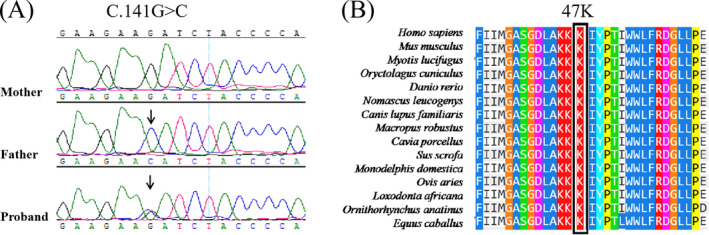
A, Sequencing results of the *G6PD* gene in all family members. Arrows indicated the position of the nucleotide changes identified in this study. The proband carried a heterozygote for c.141G > C variant in G6PD; her father was a hemizygote for c.141G > C variant, but her mother did not have the variant. B, Multiple sequence alignment showed that Lys47 was positioned in a highly conserved region. Changes in amino acids are highlighted in black boxes

The direct sequencing analysis of the exons and intronic flanking regions of the *G6PD* gene of the proband showed a heterozygous novel DNA variant c.141G > C (NM_001042351.2), which was inherited from her father (Figure 1A). This novel variant was localized in exon 3 and induced the lysine 47 asparagine (Lys47Asn) amino acid change. To the best of our knowledge, the p.Lys47Asn variant has not been reported and also absent in the databases such as Exome Aggregation Consortium (ExAC), 1000 Genomes (1000G), Single Nucleotide Polymorphism Database (dbSNP), and Human Gene Mutation Database (HGMD). The G6PD amino acid sequence alignment revealed that the lysine at position 47 is highly conserved universally (Figure 1B). Using MutationTaster, the c.141G > C variant was predicted to be disease‐causing with a score of 0.9999. Moreover, the SIFT result also indicated that c.141G > C was damaging for G6PD function. In addition, according to the 2015 ACMG guidelines and the analysis results with the tools on https://varsome.com, we classified this c.141G > C variant as a pathogenic mutation.^13^

**Figure 2:**
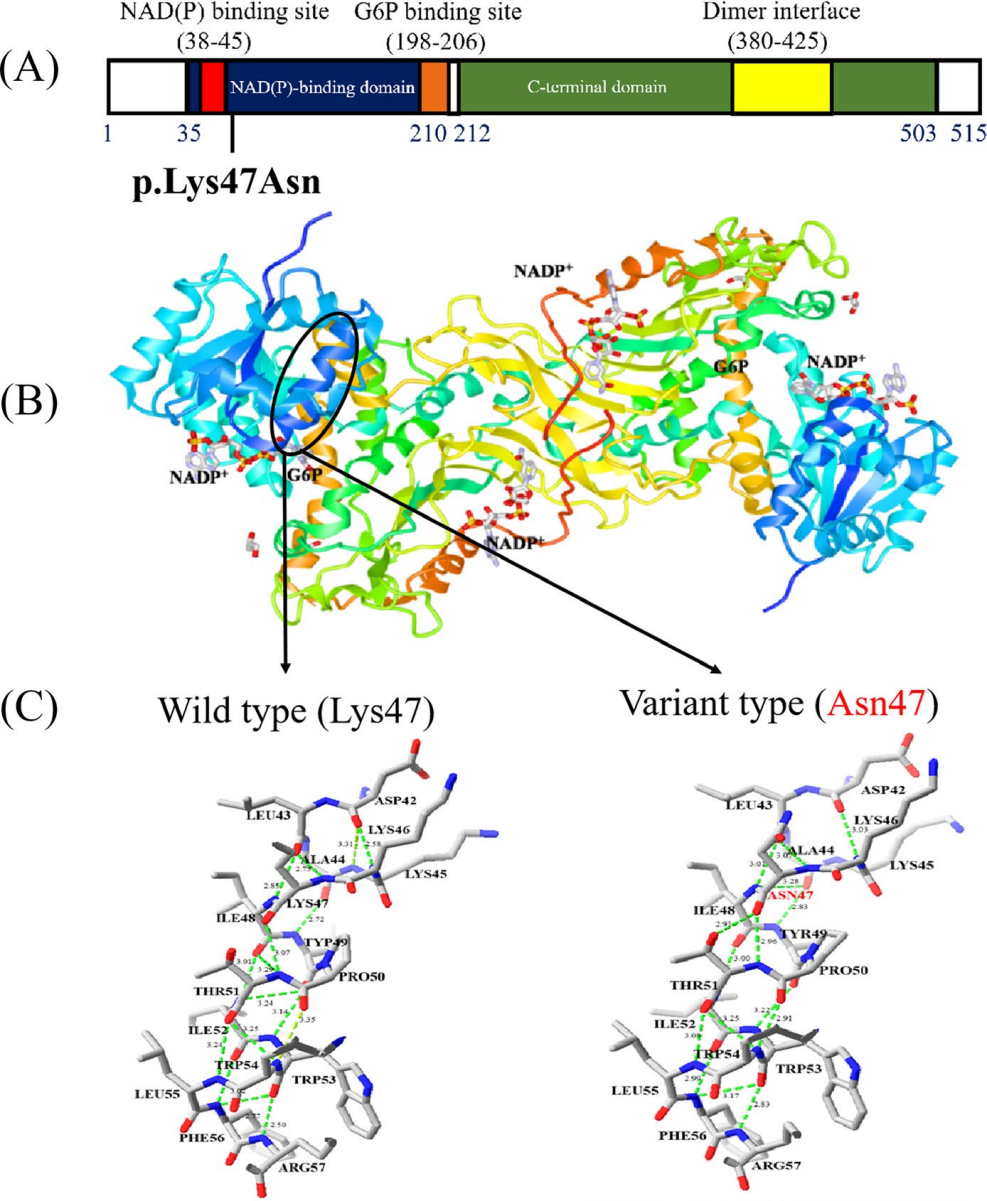
*Figure* *2* *Functional analysis of the G6PD variant protein. A, Schematic representation of alteration in the G6PD protein functional domains. The variant (c.141G > C; p.Lys47Asn) was highlighted in black in the protein domains. B, Crystallographic structure of the human G6PD enzyme, assembled from PDB:2BH9 and 2BHL. C, The hydrogen‐bonding network of wild‐type and p.Asn47 G6PD. Potential hydrogen bonds are indicated in green lines. The atoms of amino acids are colored by type (carbon is gray, oxygen is red, and nitrogen is blue)*

To analyze the impact of p.Lys47Asn change, the potential effect of this variant on the enzyme structure was analyzed. The tertiary structure model of human G6PD suggested that the mutated residue of G6PD is located in the α42‐57 helix of the G6PD molecule, which is close to the G6PD coenzyme‐binding domain (a nucleotide‐binding fingerprint GxxGGDLA, residues 38‐45 of the human enzyme) (Figure 2A and B). The nucleotide fingerprint is a conserved region, which has been associated with coenzyme binding.^14^ Analysis of the three‐dimensional structure (3D) using in silico modeling showed that Lys47 could form hydrogen bonds with residues Ile48 and Thr51 in the wild‐type G6PD (Figure 2C). However, for this variant, Lys in position 47 was replaced by Asn, while an additional hydrogen bridge was formed between the oxygen of the carbonyl group of the main chain of Asn47 and the side chain of Thr51. In addition, comparison of the model structures of the wild and mutant type showed that p.Lys47Asn variant could lead to changes in the hydrogen bonds between the subsequent residues (Figure 2C), which could contribute to the change in the molecular structure of the enzyme and the loss of catalytic activity. Taken together, as the only existing variant of the *G6PD* gene for this patient and her father, the enzyme deficiency was considered as caused by p.Lys47Asn variant. This result was also consistent with the above‐classified result.

Most G6PD‐deficient patients are usually asymptomatic; however, when exposed to situations (eating fava beans, infections, and use of certain drugs), G6PD‐deficient patients can develop acute hemolytic anemia. As the previous reports, there was also a strongly dose‐dependent manner between favism and fava bean consumption in patients with G6PD deficiency. Corresponding, acute hemolytic anemia from eating fava beans (favism) appears to much more common and more severe in children than adults.^15^ Overall, the severity of clinical phenotype in heterozygous females with G6PD deficiency is not only depended on the X‐chromosome inactivation ratio, but also with the age of the patients and dose of the trigger factors. That's why the proband, a 2.4‐year‐old heterozygous girl with *G6PD* variant (c.141G > C), had a hemolytic crisis after eating fava beans; however, her father (hemizygous G6PD‐deficient) with lower G6PD activity do not develop symptoms.

## 4 CONCLUSIONS

In this study, we describe a novel G6PD DNA variant (c.141G > C; p.Lys47Asn) in a Chinese girl with favism. This variant was located close to the G6PD coenzyme‐binding domain. Structural modeling demonstrated that the p.Lys47Asn variant could induce the change in interaction between the amino acid residues and then alter the normal G6PD protein structure. Combined with the clinical phenotype of the patient, our study demonstrated that the variant c.141G > C in *G6PD* gene was pathogenic variant in this G6PD deficiency family and could be classified as Class III (WHO classification). Our data could expand the clinical and genetic spectrum associated with G6PD deficiency. Finally, we named the c.141G > C variant as “G6PD Wuhan” based on the place of birth of the proband. This novel variant could be considered for a better comprehension of biology and molecular epidemiology of G6PD deficiency.

## CONFLICTS OF INTEREST

The authors declare that they have no conflicts of interest.

## ETHICS APPROVAL AND CONSENT TO PARTICIPATE

This study was approved according to the guidelines of the Committee on the Use of Human Subjects in Wuhan Children's Hospital.
